# Pitfalls in the Diagnosis of Coeliac Disease and Gluten-Related Disorders

**DOI:** 10.3390/nu12061711

**Published:** 2020-06-07

**Authors:** Annalisa Schiepatti, Jessica Savioli, Marta Vernero, Federica Borrelli de Andreis, Luca Perfetti, Antonio Meriggi, Federico Biagi

**Affiliations:** 1 Gastroenterology Unit of IRCCS Pavia Institute, Istituti Clinici Scientifici Maugeri, University of Pavia, 27100 Pavia, Italy; martavernero@gmail.com (M.V.); federica.bda@gmail.com (F.B.d.A.); federico.biagi@icsmaugeri.it (F.B.); 2 Allergy and Immunology Unit of Pavia IRCCS Institute, Istituti Clinici Scientifici Maugeri, 27100 Pavia, Italy; jessica.savioli01@universitadipavia.it (J.S.); luca.perfetti@icsmaugeri.it (L.P.); antonio.meriggi@icsmaugeri.it (A.M.); 3 First Department of Internal Medicine, Fondazione IRCCS San Matteo Hospital, University of Pavia, 27100 Pavia, Italy

**Keywords:** gluten, wheat, celiac disease, wheat allergy, diagnosis, non-coeliac gluten sensitivity

## Abstract

The spectrum of gluten-related disorders (GRD) has emerged as a relevant phenomenon possibly impacting on health care procedures and costs worldwide. Current classification of GRD is mainly based on their pathophysiology, and the following categories can be distinguished: immune-mediated disorders that include coeliac disease (CD), dermatitis herpetiformis (DH), and gluten ataxia (GA); allergic reactions such as wheat allergy (WA); and non-coeliac gluten sensitivity (NCGS), a condition characterized by both gastrointestinal and extra-intestinal symptoms subjectively believed to be induced by the ingestion of gluten/wheat that has recently gained popularity. Although CD, DH, and WA are well-defined clinical entities, whose diagnosis is based on specific diagnostic criteria, a diagnosis of NCGS may on the contrary be considered only after the exclusion of other organic disorders. Neither allergic nor autoimmune mechanisms have been found to be involved in NCGS. Mistakes in the diagnosis of GRD are still a relevant clinical problem that may result in overtreatment of patients being unnecessary started on a gluten-free diet and waste of health-care resources. On the basis of our clinical experience and literature, we aim to identify the main pitfalls in the diagnosis of CD and its complications, DH, and WA. We provide a practical methodological approach to guide clinicians on how to recognize and avoid them.

## 1. Introduction

Gluten-related disorders (GRD) are a group of very common and heterogeneous conditions which improve upon a gluten-free diet (GFD) [1,2]. According to Sapone et al. [1], three broad categories of GRD can be identified: (1) immune-mediated disorders including coeliac disease (CD), dermatitis herpetiformis (DH), and gluten ataxia (GA) [3,4,5]; (2) allergic reactions, such as wheat allergy (WA) [6]; (3) non-coeliac gluten sensitivity (NCGS), a condition characterized by self-reported gastrointestinal and extra-intestinal symptoms subjectively improving upon a GFD in subjects in whom other major organic GRD have been excluded [1,2,7]. This classification is mainly based on pathophysiology, meaning that a causal role for gluten in the pathogenesis of each single disorder has been established [1]. Although this is true for CD, DH, and WA [1,3,4,6], NCGS is still a poorly defined condition in spite of the huge popularity gained in the last few years [1,2,7,8,9,10,11,12,13,14,15,16,17,18,19]. Table 1 provides a comparative overview on the main diagnostic, clinical, pathological, and epidemiological aspects of the different forms of GRD.

The diagnosis of the different forms of GRD is usually made while the patient is on a normal gluten-containing diet (GCD) and in accordance with specific diagnostic criteria [1,3,4,5,6,8,9]. Misdiagnoses of GRD may result either in overtreatment of patients wrongly started on a gluten-free diet (GFD), or in severe diagnostic delays impacting on long-term morbidity and mortality and resulting in unnecessary spending of health-care resources [20,21]. This aspect is particularly relevant for CD, a condition burdened by an increased mortality and for which a long diagnostic delay has been observed. In spite of divergence, it was suggested that the diagnostic delay could be a risk factor for developing complications in coeliac patients [22,23,24,25,26,27]. The magnitude of the problem is even increased by the fact that more and more individuals in the last years have radically changed their attitude towards gluten, by embracing a GFD in the conviction, largely supported by mass media but not by any solid scientific evidence, of a healthier lifestyle, even in the absence of CD or other conditions requiring this treatment [28,29,30,31,32,33].

The identification of diagnostic errors, classified into missed, delayed, or wrong diagnoses [34,35], is strictly dependent on the existence of specific diagnostic criteria for a certain condition. Therefore, while it is possible to delineate the most common diagnostic mistakes in CD, DH, and WA [1,3,4,5,6,8,9] and to provide tips to avoid them, the lack of standard diagnostic criteria and biomarkers for NCGS makes it hardly feasible [1,2,7]. In this review, we aim to identify the main pitfalls in the diagnosis of CD and its complications, DH, and WA. We also aim to provide a practical methodological approach to guide clinicians on how to recognize and avoid them.

## 2. Coeliac Disease

Coeliac disease (CD) is an immune-mediated chronic enteropathy triggered by ingested gluten in individuals carrying the predisposing HLA-DQ2/-DQ8 haplotypes [3,36]. CD is characterized by high prevalence in the general population (around 1%) [37,38,39], an extremely heterogeneous clinical picture [3,40,41], and an increased mortality mainly due to the development of premalignant and malignant complications [22,23,24,25,26,27,42,43]. According to major international guidelines, a certain degree of villous atrophy (VA) on duodenal biopsies and positive IgA endomysial (EmA)/tissue transglutaminase (tTg) antibodies while on a normal GCD are the mainstay for diagnosis of CD in adults [44,45,46,47].

**Figure 1:**
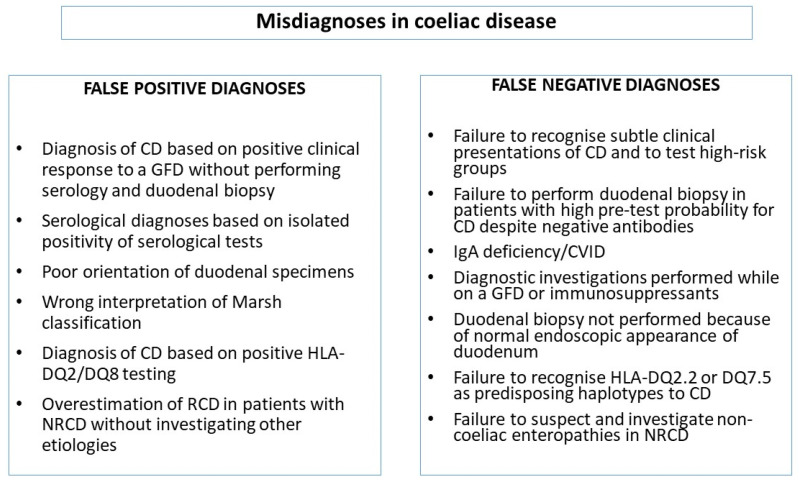
Classification of diagnostic errors in coeliac disease. CD: coeliac disease; GFD: gluten-free diet; NRCD: nonresponsive coeliac disease; RCD: refractory coeliac disease; AGA: anti-gliadin antibodies; DGP: antibodies against deamidated gliadin peptides.

Even if the diagnostic criteria for CD are very clear and the interest of patients and medical specialists in CD has been growing worldwide, the problem of diagnostic errors in CD is still remarkably relevant in clinical practice, as suggested by the large body of published papers on this topic in the last twenty years [48,49,50,51,52,53,54,55,56]. It has been reported that about 20% of coeliac patients are currently unrecognized and diagnoses are still burdened by a significant diagnostic delay [21,40,51,54,57,58,59,60,61,62]. Among patients referred to tertiary centers, CD can be misdiagnosed in as many as 30%–40% of cases [48,49]. Diagnostic errors can occur at any time during the process leading to diagnosis of CD, which includes the moment when the disease should be suspected and a diagnostic hypothesis formulated, the identification of the most appropriate diagnostic tests, and the final decision-making process based on the cognitive analysis of clinical, histological, and laboratory results. A systematic classification of the errors occurring in the diagnosis of CD is currently missing. Clinical errors in internal medicine can be generally divided into missed, delayed, and wrong diagnoses [34,35]. So, two main categories of diagnostic errors in CD can be broadly identified (Figure 1): (i) patients truly affected by CD in whom the diagnosis was not recognized or was missed or delayed (false negative diagnosis of CD); (ii) patients that had been initially diagnosed with CD and in whom, however, CD was not subsequently confirmed upon reinvestigation of their clinical picture (false positive diagnosis of CD). The entire diagnostic process of CD is to be conducted while on a gluten-containing diet, because both serological and histological findings are entirely reversible upon gluten withdrawal. Diagnostic errors can occur in any of the diagnostic steps (serology, endoscopy, histology, and HLA genetic testing) or in any combination of them.

### 2.1. Clinical Issues Leading to Misdiagnoses of CD

The most important diagnostic error is not to suspect CD in patients suffering from gastrointestinal symptoms, anemia or in conditions known to be associated to CD, especially when patients are poorly symptomatic or totally asymptomatic. Patterns of clinical presentation of CD are extremely heterogeneous and have greatly changed over the past decades. Nowadays the majority of diagnoses in adulthood are made on the basis of non-classical symptoms or in asymptomatic patients, whereas the very first cases of CD were mainly diagnosed in children presenting with frank malabsorption and failure to thrive [3,40,44,45,46,47,63]. A case-finding strategy based on Ema/tTg as the first line of investigation is the recommended diagnostic approach in patients presenting with gastrointestinal symptoms and in high-risk groups [3,44,45,46,47].

#### 2.1.1. Diagnostic Errors Related to Incorrect Timing of a Gluten-Free Diet

Consumption of gluten all throughout the diagnostic pathway leading to diagnosis of CD is mandatory in order to allow the correct interpretation of serological and histological findings [3,44,47]. A diagnostic evaluation conducted while on a GFD can lead to CD being either erroneously ruled out by negative serology or normal duodenal mucosa, or even wrongly confirmed by the clinical remission of symptoms upon a GFD [64]. Notably, an isolated clinical response to a GFD is not acceptable to confirm diagnosis of CD and may also be seen in patients with NCGS or functional disorders such as irritable bowel syndrome [2,7,10,11,12,17,19,52,64]. Unfortunately, this kind of diagnostic mistake is still very frequent in our clinical experience of tertiary referral center, since many patients have already started a GFD at the time of their first medical consultation for a suspicion of CD. In this situation, HLA typing and reintroduction of gluten into the diet are key to confirm or exclude CD [3,44,45,46,47]. More precisely, a HLA typing negative for DQ2 and DQ8 will exclude CD, while evidence of villous atrophy and positive coeliac antibodies after gluten consumption will allow a diagnosis of CD. Although a 2–8 week gluten challenge with 10 g of gluten daily has been considered the norm for a long time, it should be noted that doses and timing for gluten challenge have not been standardized yet [3,44,45,46,47,65,66,67]. In the future, possible alternatives to a gluten challenge may be offered by HLA-DQ gluten tetramer testing if this test is further validated and extended for clinical use [68].

#### 2.1.2. Serology-Related Diagnostic Errors

Several antibody tests were developed for the diagnosis of CD. They include antireticulin antibodies, anti-gliadin antibodies (AGA), antibodies against deamidated gliadin peptide (anti-DGP), EmA, and tTG [69]. According to major international guidelines on CD, in adult patients on a gluten-containing diet and displaying normal IgA immunoglobulin levels, positive IgA Ema/tTG is the mainstay for serological diagnosis of CD [44,45,46,47]. IgA human recombinant tTG antibodies are the most sensitive serological test for CD screening (sensitivity and specificity around 95%), while IgA EmA are the most specific (specificity 97%–100%) [47,69]. In children under the age of two, DGP is also reliable for serological testing, even if the ESPGHAN (European Society for Paediatric Gastroenterology and Nutrition) guidelines have recently allowed for a serological diagnosis of CD in selected children by relying on EmA and tTG as the main diagnostic tests [63]. Antireticulin antibodies are no longer in use, and AGA are obsolete and their use should be abandoned.

Serology-related diagnostic errors can occur as the consequence of using old serological testing such as AGA and DGP, or wrong interpretation of coeliac-specific serology. False positive diagnoses of CD in adults can be prompted by isolated positive AGA and isolated positive anti-DGP antibodies [47,69]. AGA were the first antibodies used for CD diagnosis, but their diagnostic role for CD is limited, given their high sensitivity but very poor specificity [44,45,46,47,69,70]. In case patients presented with positive IgA and IgG AGA, EmA and tTG are to be tested for confirmation. Similarly, anti-DGP might allow recognition of some coeliac patients that are not detected by conventional serological testing [71], but an isolated increase of these antibodies in patients with negative tTG has a very low predictive value for CD [72]. Therefore, we think that their use in the diagnosis of adult CD should be discouraged. A further possible pitfall can be represented by the methodological difference of various laboratory tests for the quantitative measurements of tTG [73].

Another serology-related diagnostic error can occur as the consequence of not taking into account assessment of concomitant IgA deficiency. False negative results for IgA EmA and tTG testing are a common cause of negative diagnostic errors in CD and may occur as a consequence of total IgA deficiency (IgA serological levels < 5 mg/dL) or in case patients had already been started on a GFD or immunosuppressants at the time of serological testing [74,75]. Prevalence of CD in IgA deficiency can be up to 7%, and nearly 2% of all the coeliac patients can have an associated IgA deficiency [76,77]. This is the reason why IgA levels must always be checked at the time of IgA tTG and EmA testing. In case IgA levels are low, IgG antibodies should be tested, and in this specific setting IgG tTG antibodies and IgG DGP were shown to have a higher sensitivity than IgG EmA [78]. In the Lombardy Region, Northern Italy, the incorporation of automatic IgA level testing together with IgA tTG has been offered since 2017 in the suspicion of CD, as suggested by ESSCD (European Society for the Study of Coeliac Disease) guidelines in 2019 [47]. We remark the old observation that IgG EmA and tTG have no relevance in diagnosing CD in the absence of IgA deficiency [78]. Presence of IgA deficiency should also prompt investigations for other causes of villous atrophy, such as giardiasis [79] and common variable immunodeficiency [80].

Finally, in patients presenting with severe intestinal malabsorption, a duodenal biopsy should be performed regardless of a negative serology [44,45,46,47,81,82,83,84]. In fact, not only malabsorption can be due to other non-coeliac enteropathies [81,82,83,85,86,87], but also up to 3%–5% of coeliac patients are affected by a form of seronegative coeliac disease (SNCD). This is a rare and still poorly defined form of CD, presenting with negative serology, classical symptoms of malabsorption, and showing clinical and histological response to a GFD [81,82,83,84,85,86,87,88]. Two Italian papers suggest a prevalence of SNCD around 2% of all the coeliac population [81,87], and it is likely that, considering different areas worldwide, the prevalence of SNCD is not higher than 5% [86]. Diagnosis of SNCD can be made only after exclusion of all the possible causes of VA and confirmed by clinical and histological improvement upon a GFD and HLA-DQ2/DQ8 positivity [81,82,83,84,85,86,87,88].

### 2.2. Endoscopy/Histology-Related Diagnostic Mistakes

Diagnostic mistakes can occur as a consequence of inadequate duodenal biopsy sampling during upper gastrointestinal endoscopy [40,89], inaccurate collection and processing of specimens resulting in poor orientation [90], and wrong interpretation of histopathological findings [40,90].

Diagnostic errors during endoscopy can lead to either underestimation or overestimation of CD. Histological lesions of CD may have a patchy distribution along the duodenal mucosa. For this reason, a minimum of four biopsies should be taken from areas with endoscopic features suggestive for CD (flattened duodenal folds, scalloping mucosa, visibility of submucosal vascular pattern, erosions) in different parts of the duodenum (from the bulb to the distal duodenum) [91,92,93]. On the other hand, a normal endoscopic appearance does not obviously exclude CD. So, duodenal sampling is mandatory any time a gastroscopy is performed in the suspicion of CD. Single-bites biopsies technique is preferable over double-bites biopsies technique, since the former allows a better orientation of duodenal specimens [94]. Current medical literature suggests that up to 13% of patients with diagnosed CD have had a previous endoscopy with inadequate biopsy sampling, thus resulting in delayed diagnosis [41,89].

According to our experience and the literature, inaccurate collection and processing of duodenal specimens is a common cause of false positive diagnosis of CD [48,90,95,96]. This is very often due to poor orientation of duodenal specimens, which causes partial fusion or crushing of adjacent villi to be misinterpreted as frank villous atrophy [90,95]. It is also possible that a flat mucosa can be misinterpreted as normal as a consequence of poor orientation, thus leading to a false negative diagnosis. This is a rare event due to the cross-section of the crypts in misoriented and tangentially cut mucosal specimens [90].

Correct orientation of duodenal samples is, therefore, highly recommended in order to avoid misinterpretation of duodenal histology, particularly when histological evaluation is performed by pathologists with insufficient expertise on CD or gastrointestinal disorders [90,95]. Orientation should be performed using cellulose acetate filters that ensure the perfect adhesion of the sample to the support and correct processing of the sample during fixing and cutting phases [95].

Finally, wrong interpretation of duodenal histopathological findings can often lead to false positive diagnoses of CD, which are mainly due to misinterpretations of the grading of histological lesions provided by the Marsh classification [48,55,97,98,99,100]. This can be due to different causes. Firstly, inter-observer variability of Marsh classification can play a role and for this reason Corazza–Villanacci histological grading has been suggested [98]. Recently, we have shown that in everyday clinical practice a classification of the histological grading is not necessary for the diagnosis of CD and other non-coeliac enteropathies [99]. Secondly, although VA, crypth hyperplasia, and raised intraepithelial lymphocyte count are the histological hallmarks of CD, these lesions are not specific to CD and can be found also in other conditions [81,82,85,97,101]. However, in patients with positive EmA/tTG and architecturally normal duodenal mucosa, a diagnosis of potential CD can be made and furtherly supported by HLA typing showing DQ2 and/or DQ8 molecules [102,103]. Potential CD is a form of CD that can be found in up to 18% of coeliac patients [102].

### 2.3. Diagnostic Errors Related to HLA Typing

Although HLA-DQ2 and DQ8 molecules represent the most important genetic factors in the development of CD, HLA typing is not necessary for the routine diagnosis of CD [44,45,46,47], provided that the correct diagnostic pathway is fulfilled. The majority of CD patients (90%–95%) carry HLA-DQ2.5 molecules (encoded by DQA1*05 and DQB1*02 alleles), whereas the remaining 5%–10% of patients carry either HLA-DQ8 (encoded by DQA1*03 DQB1*03:02) or HLA-DQ2.2 (encoded by DQA1*0201 DQB1*0202). Finally, few patients (<1%) not carrying these heterodimers express the HLA-DQ7.5 molecules (DQA1*05 DQB1*0301) [36,104]. Nearly 30%–40% of the general population carry at least one of these haplotypes, therefore a positive HLA typing is not sufficient on its own for making a diagnosis of CD. It only indicates the possibility for a patient to develop CD. On the other hand, a negative testing for HLA DQ2 and DQ8 has a negative predictive value of nearly 100% [36,104]. However, in our clinical experience and the reported literature, false positive diagnoses of CD can occur on the basis of a genetic test showing HLA DQ2.5 or HLA-DQ8 molecules [53]. Only rarely a true diagnosis of CD was missed because of wrong interpretation of HLA-DQ2.2 or HLA-DQ7.5. Therefore, in clinical practice, HLA typing should be requested only if results of serology and histology do not allow a definitive diagnosis of CD or in particular forms of CD that include potential CD, seronegative CD, and complicated CD [25,26,27,82,83,85,86,87,88,97,102,103].

## 3. Non-Responsive Coeliac Disease

**Figure 2:**
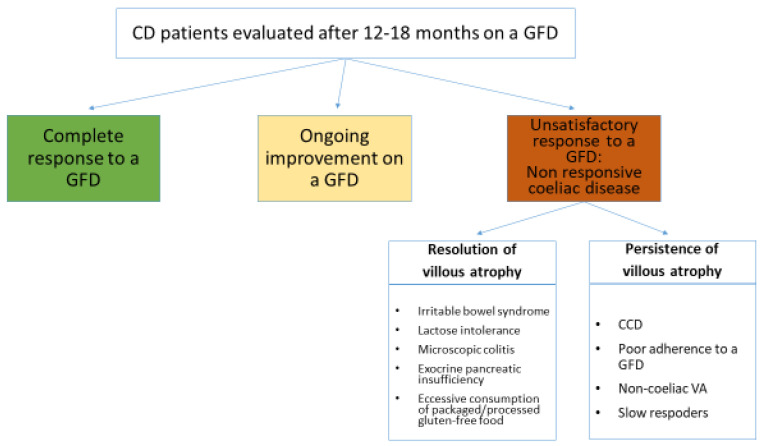
Proposal for a nosographic classification of clinical scenarios responsible for persisting clinical and/or histological abnormalities in coeliac patients on a gluten-free diet for 12–18 months. GFD: gluten-free diet; VA: villous atrophy; CCD: complicated coeliac disease.

Nonresponsive coeliac disease (NRCD) has been defined as the persistence of symptoms, signs, laboratory abnormalities, or histological changes typical of CD despite adherence to a GFD for 6–12 months [105,106]. We agree with this definition by Penny et al. and we also agree on their statement that in this definition it is necessary to provide a timeframe, but this is inevitably arbitrary. Not only the kinetics of clinical, histological, and serological response to a GFD is highly variable among coeliac patients, but also in the same patient the normalization of clinical, histological, and serological findings can occur at different time points [106]. Since in the Oslo classification a definition of NRCD was not provided [107], we consider the term NRCD as an “umbrella term” referring to variegated clinical scenarios, all characterized by an unsatisfactory clinical/histological response to a GFD, but totally different prognoses. Figure 2 is an attempt of a nosographic classification of all these scenarios. The concept of NRCD does not include those coeliac patients on a strict GFD who have been starting to improve both clinically and histologically since diagnosis, but in whom a full recovery of clinical/laboratory signs, symptoms, and histology has yet to be obtained at time of first follow-up evaluation. Although both advertent and inadvertent dietary lapses are the most common cause of NRCD [105,106], it should be noted that complete resolution of symptoms and histological lesion is achieved only after many years on a strict GFD and this is crucial to prevent long-term poor outcomes and complications. Therefore, the lack of a complete resolution of clinical signs and histology is likely to be due to follow-up examinations performed too early and it should not be taken for NRCD.

The prognosis of the conditions listed in Figure 2 is extremely variable. For some of them it is very good and they only need to be recognized to avoid unmotivated concerns and useless investigations. This is the case for coeliac patients with associated irritable bowel syndrome or lactose malabsorption [105,106]. On the other hand, the prognosis of the other conditions such as complications of CD and VA unrelated to gluten consumption is very poor [23,24,25,26,27,81,82,85]. 

In our clinical experience, several diagnostic errors can occur in coeliac patients on a GFD. The first one is the overestimation of cases of NRCD and refractory coeliac disease (RCD) in those patients who have been starting to improve on a GFD, but in whom a complete response has yet to be reached at time of first follow-up. The second type of error is related to the overestimation of RCD in patients with NRCD. This mainly occurs as the consequence of erroneously considering NRCD as synonymous of refractory CD, which is instead characterized by persistent malabsorption and villous atrophy despite a strict GFD for at least 12 months [107,108]. This aspect is confirmed by old data from literature stating that up to 20% of coeliac patients did not show a satisfactory response to a GFD [109]. More recently, after having established that the main criterion for defining the refractory state is the lack of histological response to a GFD, prevalence of RCD has been reported to be around 1% [25,110,111,112,113]. Another aspect which is worth mentioning is that in a large proportion of cases persisting symptoms in coeliac patients depends on ongoing gluten ingestion or other conditions unrelated to CD such as irritable bowel syndrome, pancreatic insufficiency, lactose intolerance, or microscopic colitis [105,106]. 

The third type of error is related to misdiagnosing RCD in patients with severe villous atrophy and malabsorption due to enteropathies unrelated to gluten ingestion such as autoimmune enteropathy, common variable immune-deficiency, olmesartan-associated enteropathy, and idiopathic villous atrophies [81,82,85,87,97,114,115].

We conclude that in coeliac patients on a GFD still suffering from persisting symptoms, a specific and thorough investigational workout is mandatory not only to ascertain the real clinical significance of these persisting symptoms, but also to exclude dietary lapses and other concurrent medical conditions. Finally, in patients with confirmed lack of histological response to a strict GFD, the initial diagnosis of CD needs to be reinvestigated and the possibility of a complication of CD or a non-coeliac enteropathy carefully investigated [3,44,45,46,47,105,106].

## 4. Dermatitis Herpetiformis

Dermatitis herpetiformis (DH) is an extra-intestinal manifestation of CD (estimated prevalence of 30–75 per 100,000), characterized by itchy and blistering polymorphic rash typically involving the elbows, knees, and buttocks which is reversible upon a GFD in the vast majority of cases [4,44,45,46,47,62,116,117]. Gastrointestinal symptoms and family history of CD may or may not be part of the clinical picture at diagnosis. The gold standard for diagnosis of DH is detection in immunofluorescence of granular IgA deposits in the dermal papillae of perilesional skin biopsy while the patient is on a normal gluten-containing diet. According to literature, false negative immunofluorescence results occur in about 5% of the patients, especially if the biopsy has been taken from blisters or inflamed skin or if the patient has already been started on a GFD [118,119]. Considering that negative tTG and normal intestinal biopsies can be found in up to one-third of patients with DH, a negative antibody testing is not sufficient to rule out the possibility of DH, and if clinical suspicion of DH is high, a skin biopsy needs to be performed. HLA determination should only be applied in obscure cases, and if performed, a HLA DQ2 and DQ8 negative result rules out DH and CD [44,45,46,47,104]. A duodenal biopsy is not always necessary in patients with DH diagnosis confirmed by skin biopsy [4,44,45,46,47]. However, if severe gastrointestinal symptoms or anemia are present at diagnosis, then a duodenal biopsy is to be performed. GFD should be strict and life long, and dietary adherence offers an excellent long-term prognosis [4,120,121].

## 5. Wheat Allergy

Wheat allergy (WA) is defined as an adverse immunologic response to wheat proteins that can result in different clinical manifestations, depending on the route of allergen exposure and the type of immunological reaction involved (IgE mediated or non-IgE mediated) [6,122,123]. IgE-mediated reactions to wheat can occur after either ingestion (food allergy) or inhalation (respiratory allergy). Clinical manifestations include urticaria/angioedema, acute gastrointestinal symptoms (vomiting, abdominal pain, diarrhea), acute exacerbation of atopic dermatitis and wheat-dependent exercise-induced anaphylaxis (WDEIA). Respiratory WA mainly refers to occupational asthma (or baker’s asthma) and rhinitis [6,122,123,124,125]. Among the non-IgE-mediated forms, eosinophilic esophagitis and eosinophilic gastritis have been described to be triggered by wheat [6,126,127].

Food allergies are increasingly recognized as a growing health issue worldwide. However, the true prevalence of WA diagnosed by oral food challenge is still undefined. Children are more commonly affected than adults [122,128,129]. On the basis of our clinical experience and the current literature [122,128,130], the self-reported prevalence of WA largely overestimates the true prevalence assessed by oral food challenge. Although prognosis of WA is usually favorable, particularly in children outgrowing their food allergies, some serious and life-threatening events may occur, such as severe anaphylaxis [131]. Therefore, correct diagnosis of WA and its subtypes is mandatory not only to prevent long-term complications, but also to deliver the most appropriate treatment. In this regard, it should be noted that even though WA is considered in the spectrum of GRD, its dietary management relies on avoidance of wheat in both alimentary and non-alimentary products. This is not equivalent to a GFD. Cereals containing prolamins, such as barley, rye, and oat, that must be avoided by coeliac patients on a GFD, are generally well tolerated in WA. Conversely, gluten-free food may contain wheat starch in traces and therefore may not be indicated in WA. Strictness of the dietary treatment, however, depends on the type and severity of clinical manifestations and should be guided by an expert dietician [132]. For example, while for patients with food allergy it is important to prevent also respiratory exposure to wheat dust, in respiratory allergy it is not necessary to avoid wheat ingestion. This is due to the different epitopes involved: conformational epitopes in respiratory allergy, which can be inactivated by cooking or digestion, and linear epitopes in food allergy, which are unmodified by heat or acid [133].

### 5.1. IgE-Mediated Wheat Allergy

Diagnosis of WA is usually based on a combination of specific IgE to wheat via skin prick tests (SPT) or specific IgE measurement (sIgE) and oral food challenge (OFC). The first step in the diagnostic pathway is a detailed clinical history documenting type of symptoms and their onset (within 1–3 h after the exposure), amount and form of allergen ingested (raw, cooked, semicooked, baked), and associated factors (physical exercise, alcohol consumption, drugs such as non-steroidal anti-inflammatory drugs). In patients presenting with such symptoms, skin prick test (SPT) and in vitro specific immunoglobulin E assay (sIgE) are the first-level diagnostic tests in the suspicion of WA [6,122,123,134,135]. However, the diagnostic accuracy of SPT and sIgE is still suboptimal. SPTs are affected by low sensitivity, because commercial test reagents are mixtures of water/salt-soluble wheat proteins that lack allergens from the insoluble gluten fraction. In vitro sIgE assays are more sensitive but less specific because of frequent cross-reactivity with grass pollens due to shared IgE epitopes [136,137,138]. 

Molecular-based allergy diagnostics can identify specific wheat allergens and their potential association with clinical manifestations of WA [1,3,12]. However, sensitivity and specificity of these tests are still unsatisfactory and their role in clinical practice is still limited. In particular, some allergens seem mainly associated with respiratory symptoms (alpha- amylase/trypsin inhibitor), food allergy (nonspecific lipid-transfer protein, gliadins), WDEIA (ω-5 gliadin), or contact urticaria (high-molecular-weight glutenins) [6,123,136]. Molecular-based allergy diagnostics can be helpful to identify patients with WDEIA (association in 80% of patients) and to discriminate between baker’s asthma and cross-reaction with pollens [6].

Functional assays are still considered the gold standard for diagnosis [137,138]. These tests include elimination diet (4–8 weeks), followed by a possible planned re-challenge test (open, single-, or double-blind placebo-controlled food challenge) or initial food reintroduction procedure [139]. In baker’s asthma, bronchial challenge test (inhalation of increasing doses of allergenic extracts) has to be performed [140].

Basophil Activation Test (BAT) is a further diagnostic test based on flow cytometry, which has progressively started to move from the research setting to clinical practice [141]. BAT has been studied in the diagnosis of a variety of food allergies, including WA. Although sensitivity and specificity of BAT for wheat proteins are still suboptimal [142], BAT has been proposed as a second diagnostic test before proceeding to OFC in selected cases where clinical history is suggestive for WA, but SPT and sIgE give equivocal results [143]. However, feasibility and cost-effectiveness of BAT in multiple real-life clinical scenarios still need to be confirmed on a large scale [144].

In our clinical experience, a major pitfall in the diagnosis of WA is given by the incorrect use of SPT and sIgE and/or by the misinterpretation of their results in patients presenting with a low pretest probability of having WA. In other words, the isolated presence of specific SPT and sIgE without a clear history of symptoms following wheat exposure is not diagnostic of WA. A positive test without specific symptoms may indicate only that these patients are sensitive to wheat, but they can however tolerate wheat exposure, as demonstrated in grass pollen-sensitive individuals [145]. Similarly, increased concentrations of sIgE and the size of the SPT wheal are just associated with the likelihood of a clinical reaction, but they are not predictive of its severity [122,146]. Therefore, the methodological approach to a correct diagnosis of WA is to request these diagnostic tests only if the pretest probability for an allergy is high, so as to avoid making false positive diagnoses of WA.

### 5.2. Non-IgE-Mediated Wheat Allergy

Wheat has been found to be an important trigger of some eosinophilic disorders of the gastrointestinal tract developing largely as a consequence of non-IgE-mediated mechanisms [126,147,148]. They include eosinophilic esophagitis (EE) and gastritis (EG) [126,127,147], whereas the pathogenetic role of wheat in eosinophilic enteritis and colitis is still unclear. EE is the prototype of these disorders and can develop in both children and adults [127,147,148]. Wheat is the second most common allergen after milk, able to trigger EE in up to 12% of children [139] and up to 30%–60% of adults affected by this disorder [149,150].

Diagnosis of EE is clinical and histopathological, being based on persistence of esophageal symptoms (dysphagia, food impaction, vomiting, reflux) unresponsive to at least 8 weeks of proton-pump inhibitors therapy and on typical histological findings (≥15 eosinophils per high power field on esophageal biopsies taken during gastroscopy) [148,149,150]. SPT and sIgE for wheat have little sensitivity and specificity and have no role in predicting a possible response to a wheat elimination diet [147]. Therefore, elimination diet still remains the gold standard for identifying the role of a food allergen in triggering EE.

Eosinophilic gastroenteritis is a rare and still poorly defined condition for which standard diagnostic criteria are still lacking. In our experience, it is not uncommon to see false positive diagnoses of eosinophilic gastroenteritis based on histological findings on a slightly increased number of eosinophils.

## 6. Non-Coeliac Gluten Sensitivity

The term NCGS has been introduced referring to individuals who self-report gastrointestinal and extra-intestinal symptoms subjectively believed to be induced by the ingestion of gluten-containing food and in whom CD and its complication, WA, and other major organic intestinal and extra-intestinal disorders, have been thoroughly excluded. In these patients, remission of symptoms occurs upon a GFD [1,7,10,11,17,19,47].

Although the first description of NCGS dates back to 1980 [151], it is only in the last years that the concept of self-reported NCGS has gained prominence [1,2,7,152,153]. This has certainly been fueled by a radical change in the attitude towards gluten by many people considering a GFD as a choice for a healthier lifestyle, despite the absence of medical conditions requiring this treatment [28,29,30,31,32,33,152]. This aspect together with the current lack of specific diagnostic criteria and biomarkers for NCGS, and the possible overlap with functional disorders make the estimation of the worldwide prevalence of this condition and the identification of diagnostic errors virtually impossible [1,2,7,129]. On the other hand, it is extremely common to see patients who have already started a GFD seeking medical consultation in the suspicion of CD, WA, and other disorders unrelated to gluten ingestion. While a GFD has no effect on the diagnostic pathway of many extra-intestinal disorders, on the contrary it represents a massive impediment to a correct diagnosis of CD, DH, and WA, as already discussed.

The most common reported gastrointestinal symptoms in NCGS are bloating (87%), abdominal pain (83%), diarrhea (54%), epigastric pain (52%), nausea (44%), and aerophagia (36%). Extra-intestinal manifestations include lack of well-being (68%), tiredness (64%), headache (24%), anxiety (39%), “foggy” mind (38%), and joint/muscle pain compatible with fibromyalgia (31%); in a small group of patients some allergic manifestations, such as asthma or rhinitis, are also described [1,2,7,153]. Recently, the association of NCGS with psychiatric and neurological conditions, such as autism, ataxia, epilepsy, and mood disorders, has been reported [5,8,9,154]. Although these gastrointestinal and extra-intestinal symptoms occur relatively soon—in few hours or days—after the ingestion of gluten-containing products and vanish upon withdrawal of gluten, they are not specific for NCGS. Therefore, the clinical suspicion of NCGS can be considered only in individuals in whom CD and its complications, WA, and other major organic extra-intestinal disorders have been excluded by following a rigorous methodologic diagnostic approach. At this point diagnosis of NCGS should be confirmed by performing a double-blind, placebo-controlled challenge [1,2,7,10,11,17,19]. To make a clear distinction between NCGS and irritable bowel syndrome is rather difficult, and the possibility of an overlap between these two conditions should be considered. Therefore, either a GFD or a low FODMAP (fermentable oligo-, di-, mono-saccharides and polyols) diet may be considered in these patients [12,13,16,18].

## 7. Conclusions

CD and WA are common conditions for which misdiagnoses are still commonly encountered in clinical practice. Before starting patients on a GFD it is mandatory to reach a final diagnosis to avoid overtreatment and unnecessary health care expenditure. Compliance with international guidelines and adoption of a methodological diagnostic approach while the patient is on a normal gluten-containing diet are required to reduce diagnostic delay and diagnostic errors.

## Figures and Tables

**Table 1:** Comparative overview on clinical, pathological, and epidemiological features of the different types of gluten-related disorders.

	Coeliac Disease	Dermatitis Herpetiformis	Gluten Ataxia	Wheat Allergy	NCGS
**Prevalence in the general population**	- ≈1% - Upward trend in the last decades	30–75 per 100,000	- unknown - GA accounts for up to 40% of idiopathic ataxias	Prevalence assessed by OFD still unknown	- **Unknown** - **Supposed to be higher than in CD**
**Pathogenesis**	- Predominant adaptive restricted HLA-DQ2/DQ8 immune response to gluten - Role of TG2	Role of TG3 enzyme	AGA cross-react with epitopes on Purkinje cells	- IgE-mediated - non-IgE mediated	- Unknown - Role of innate immune response?
**Genetics**	HLA-DQ2 and DQ8 restricted	HLA-DQ2 and DQ8 restricted	Not HLA restricted	Not HLA restricted	Not HLA restricted
**Serum antibodies**	- IgA tTG/EmA^+ve^ - IgG tTG/EmA^+ve^ if IgA deficiency - true SNCD is rare	- tTG3 ^+ve^ - IgA tTG/EmA ^+ve^ in 70%–75% of patients	- tTG 6 ^+ve^ antibodies - AGA IgA/IgG	- positive serum IgE to wheat	- **tTG/EmA^-ve^** - **IgG AGA+ve?** - **Lack of specific serological markers**
**Small bowel histology**	- duodenal VA is the hallmark - normal duodenal architecture only in PCD	Increased IEL in almost 100% but frank VA in only 70%–75% of patients	Duodenal VA in up to 40% patients	Normal duodenal histology	Normal duodenal architecture
**Clinical** **picture**	- Classical: frank malabsorption - Non-classical: extra-intestinal symptoms and/or associated conditions - Silent: asymptomatic patients, mainly detected by screening	Itchy blistering rash involving elbows, extensor surfaces of forearms, knees and buttocks	- gait and lower limb ataxia ± other GI or extra-GI symptoms	Intestinal and extra-intestinal symptoms within minutes to 1–3 h after exposure to wheat	Intestinal and extra-intestinal symptoms
**Risk of complications**	- Increased (classical symptoms and age at diagnosis >40) - CCD includes: RCD1, RCD2, EATL, SBC, BCL	Not increased	Progression of neurological dysfunction	Increased (anaphylaxis)	**Unknown**
**Morbidity**	Increased	Not increased	Increased	Increased	**Unknown**
**Mortality**	Increased	Not increased	Increased	Increased	**Unknown**

Grey color indicates areas of uncertainty. CD: coeliac disease; SNCD: seronegative coeliac disease; PCD: potential coeliac disease; CCD: complicated coeliac disease; RCD1: refractory coeliac disease type 1; RCD2: refractory coeliac disease type 2; EATL: enteropathy associated T-cell lymphoma; BCL: B-cell lymphomas; SBC: small bowel carcinoma; GA: gluten ataxia; NCGS: non-coeliac gluten sensitivity; OFD: oral food challenge; TG2: tissue tranglutaminase type 2; TG3: tissue transglutaminase type 3; EmA: endomysial antibodies; tTG: tissue transglutaminase antibodies; GI: gastrointestinal; AGA: anti-gliadin antibodies; VA: villous atrophy; IEL: intraepithelial lymphocytes.
